# Extracellular Matrix in Calcific Aortic Valve Disease: Architecture, Dynamic and Perspectives

**DOI:** 10.3390/ijms22020913

**Published:** 2021-01-18

**Authors:** Anna Di Vito, Annalidia Donato, Ivan Presta, Teresa Mancuso, Francesco Saverio Brunetti, Pasquale Mastroroberto, Andrea Amorosi, Natalia Malara, Giuseppe Donato

**Affiliations:** 1 Department of Experimental and Clinical Medicine, University Magna Graecia of Catanzaro, 88100 Catanzaro, Italy; tmancuso@unicz.it (T.M.); mastroroberto@unicz.it (P.M.); nataliamalara@unicz.it (N.M.); 2 Department of Health Sciences, University Magna Graecia of Catanzaro, 88100 Catanzaro, Italy; annalidia.donato@gmail.com (A.D.); presta@unicz.it (I.P.); francescosaverio.brunetti@studenti.unicz.it (F.S.B.); amorosi@unicz.it (A.A.); gdonato@unicz.it (G.D.)

**Keywords:** calcific aortic valve disease, extracellular matrix, extracellular vesicles, collagen, elastic fibers, tenascin-C, periostin

## Abstract

Calcific Aortic Valve Disease (CAVD) is the most common valvular heart disease in developed countries and in the ageing population. It is strongly correlated to median age, affecting up to 13% of the population over the age of 65. Pathophysiological analysis indicates CAVD as a result of an active and degenerative disease, starting with sclerosis and chronic inflammation and then leaflet calcification, which ultimately can account for aortic stenosis. Although CAVD has been firstly recognized as a passive event mostly resulting from a degenerative aging process, much evidences suggests that calcification arises from different active processes, involving both aortic valve-resident cells (valve endothelial cells, valve interstitial cells, mesenchymal stem cells, innate immunity cells) and circulating cells (circulating mesenchymal cells, immunity cells). Moreover, a role for the cell-derived “matrix vesicles” and extracellular matrix (ECM) components has also been recognized. The aim of this work is to review the cellular and molecular alterations occurring in aortic valve during CAVD pathogenesis, focusing on the role of ECM in the natural course of the disease.

## 1. Calcific Aortic Valve Disease

Calcific aortic valve disease (CAVD) is the most common valvular heart disease in developed countries and in the ageing population [1]. Among risk factors, such as hypertension, smoking, diabetes mellitus, elevated cholesterol, and male gender, genetic and developmental origins have also been identified. For example, bicuspid aortic valve (BAV) is a common congenital heart valve disease that may lead to CAVD via the dysregulation of RUNX2 expression [2,3,4]. The prevalence of CAVD is strongly associated to median age, affecting up to 13% of the population over the age of 65 [5]. At present, surgical valve replacement represents the standard treatment option. For patients presenting contraindications to the classic surgical approach, trans-catheter aortic valve replacement (TAVR) constitutes the treatment of choice [6].

Pathophysiology of CAVD includes a disease continuum from sclerosis to chronic inflammation and then leaflet calcification, which ultimately can account for aortic stenosis (AS). CAVD was for a long time considered a degenerative disease, with a gradual accumulation of the calcium in the valve leaflets. It is now clear that it is instead the result of some active and complex cellular processes, firstly coordinated by valve interstitial cells (VICs), vascular endothelial cells (VECs) and inflammatory cells, which account for extracellular matrix (ECM) reorganization.

In this work, the authors provide an extensive review of the literature concerning the role of ECM in the establishment of the two major hallmarks of CAVD: fibrosis and mineralization.

## 2. An Overview of the Cellular Processes Involved in Aortic Valve Calcification

In humans, the aortic valve is an avascular structure consisting of three leaflets supported by a fibrous skeleton. Each leaflet is <1 mm in thickness and is comprised of three distinct ECM layers including the *fibrosa*, *ventricularis* and *spongiosa*, covered by an outer (or aortic) and an inner (or ventricular) layer of endothelial cells, defined as vascular endothelial cells (VECs) [7]. The three ECM layers are enriched in VICs, derived from the cardiac neural crest [8]. VICs are mostly recognized as quiescent fibroblasts which, in specific physiological or pathological conditions can be activated, accounting for remodeling of the ECM. Moreover, smooth muscle cells could also be identified mainly at the base of the *ventricularis* [9,10].

As pointed out by Ma X et al., human quiescent VICs are responsible for maintaining production/degradation balance of ECM components [11]. Following specific stimuli, such as mechanical or chemical stress of the endothelium, human quiescent VICs differentiate in myofibroblasts expressing α-smooth muscle actin (α-SMA), able to regulate valve remodeling. Fibroblasts–myofibroblasts differentiation is reversible, so that there exists a calibrate balance between quiescent and activated VICs. Generally, human aortic valves show a low number of myofibroblasts, precisely ~20% for neonatal, ~6% for child and ~2.5% for adult valves [12]. In particular conditions, VICs can also acquire an osteoblast-like phenotype [7,13,14], via the reactivation of bone morphogenetic protein (BMP)-induced Wnt/β–catenin signaling and transcription factor Msh Homeobox 2 (Msx2) [8]. Aortic valve osteoblasts account for calcium crystal organization in structures similar to lamellar bone, that represents about 15% of the calcified mass of the valve. Moreover, during CAVD pathogenesis, VIC-derived myofibroblasts or osteoblasts account for the microcalcification process, which is responsible for about 85% amorphous diffuse calcified mass in the valve [15]. According to the data of [16], human calcified aortic valves also host osteoclasts, however they appear to favor calcification in some way.

Similarly, VECs can also differentiate in osteoblasts via a process of endothelial–mesenchymal transition (EndMT). Briefly, as observed in VIC activation, VEC differentiation results from several pathological conditions, such as oscillatory shear stress and inflammatory cytokines [17]. Upon stimulation, VECs progressively lose their endothelial cell phenotype and activate myofibroblast gene regulatory programs. Following the acquisition of a mesenchymal or myofibroblast phenotype, osteogenic differentiation can also occur [17].

Physiological presence of mesenchymal stem cells in the aortic valve has also been shown, as well [18]. Therefore, both VECs and VICs as well as mesenchymal stem cells populating normal aortic valves can differentiate in osteoblasts, contributing to the calcification process.

## 3. The Role of Inflammation and Oxidative Stress in the Onset of Aortic Valve Calcification

Although for a long time CAVD pathogenesis has been described as a degenerative process with passive calcium deposition, recently many active processes have been implicated in the progressive calcification of the aortic valve. Two mechanisms have been mainly recognized as triggering factors: mechanical stress of endothelium, and lipid deposition. Prolonged exposure to these conditions accounts for the activation of biological cascades which are ultimately responsible for the inflammatory reaction and high level of oxidative stress. The relative contribution of lipid deposition and endothelial injury in inflammation exacerbation has not been established yet, and crosstalk mechanisms may also be involved [19,20].

The idea that oxidative stress and above all the oxidation of low-density lipoprotein (LDL) might play a role in CAVD originated at least two decades ago [21]. Subsequently, the involvement of oxidative stress has been investigated via both in vitro and in vivo studies [22,23]. Today, although the association between the accumulation of lipids in the aortic valve and the progression of calcification has been widely accepted, the characterization of the specific pathways involved in such a process is still at the beginning. Indeed, the different active biomolecules produced by the enzymatic reaction of accumulated lipids are still under investigation [24]. Many difficulties also derive from the heterogeneous composition of oxidized low-density lipoproteins (Ox-LDLs) and oxidized phospholipid (Ox-PL) detected in calcified aortic valve [25]. Recently, studies have shown that the High-Density Lipoprotein (HDL), widely recognized for its protective role from oxidation and inflammation, might have a role in CAVD pathogenesis [26,27]. In particular, a high level of ox-HDL has been reported in the plasma of CAVD patients. Additionally, the administration of ox-HDL in VICs culture accounts for mineral deposit production, showing a direct relationship between ox-HDL and osteogenic differentiation of VICs [26]. Although VICs are indicated as the major driver of oxidative stress in CAVD, VECs seem to be also involved [28].

**Figure 1:**
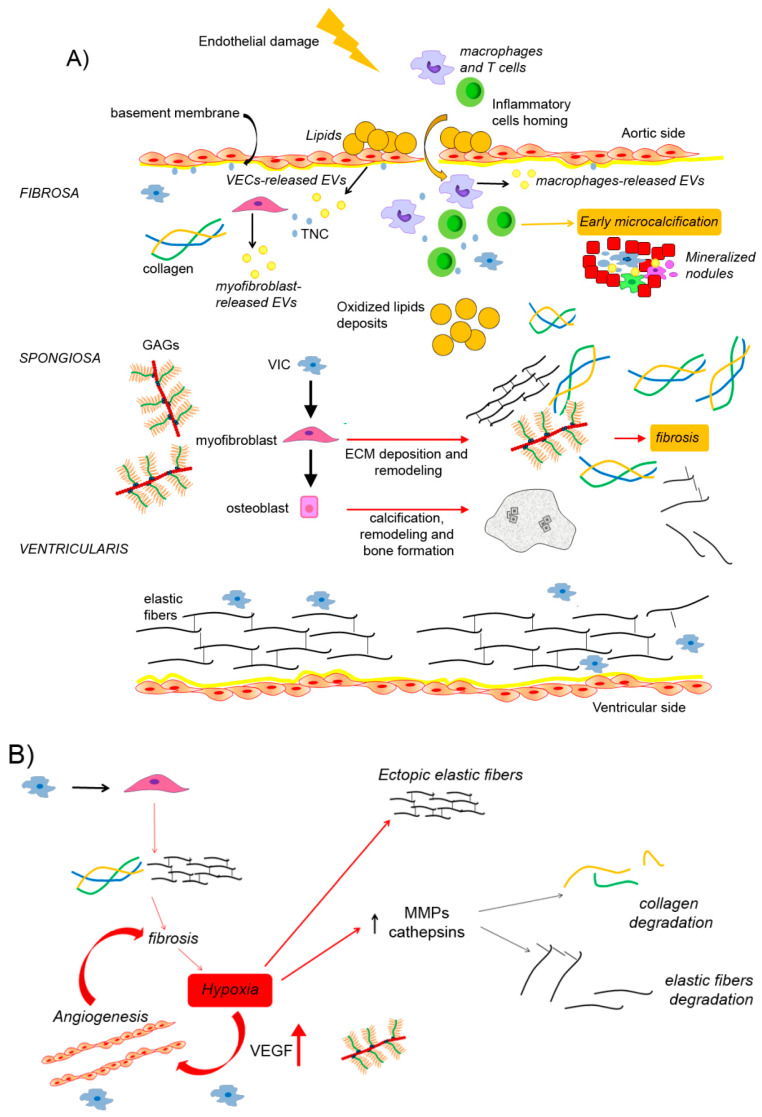

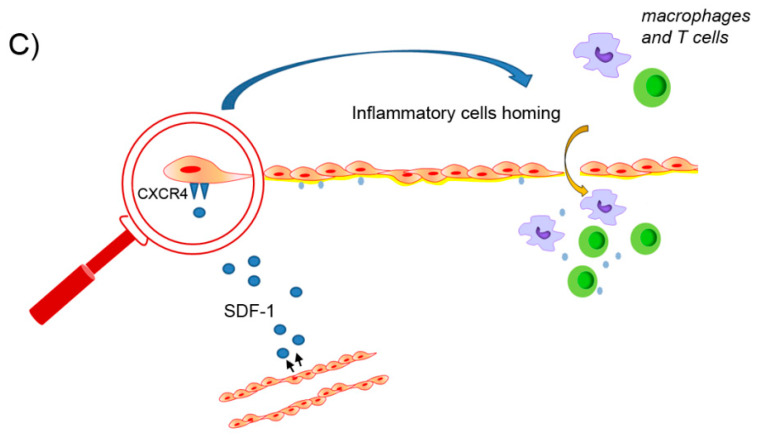
Schematic representation of Calcific Aortic Valve Disease (CAVD) pathogenesis. Collagen is the predominant component of the *fibrosa* in healthy cardiac valves, while the *spongiosa* and *ventricularis* are mainly composed of proteoglycans (PGs) and a collagen/elastin network, respectively. (**A**) Initial lesions, such as endothelial damage, account for the recruitment of inflammatory elements in the valve, triggering initial microcalcification. Resident cells such as VICs and VECs as well as circulating macrophages support the calcification process via the release of extracellular vesicles. Apoptotic myofibroblasts and immunity cells favor nucleation of calcium and phosphate. The resulting inflammatory microenvironment and myofibroblasts differentiation cause extracellular matrix (ECM) remodeling, valve thickening and fibrosis. Further differentiation in osteoblasts accounts for calcification, remodeling and biomineralization or ossification. Ectopic expression of TNC is also depicted. (**B**) Hypoxic conditions induce the formation of new ectopic vessels which in turn support ECM remodeling via MMP- and cathepsins-dependent degradation of collagen and elastic fibers, respectively. (**C**) The activation of the SDF-1/CXCR4 pathway in endothelial cells further stimulates the homing of inflammatory elements (**C**). CXCR4, C-X-C Motif Chemokine Receptor 4; EVs, extracellular vesicles; GAG, glycosaminoglycans; SDF-1, stromal cell-derived factor 1; TNC, tenascin-C; MMPs, matrix metalloproteinases; VECs, valve endothelial cells; VEGF, vascular endothelial growth factor; VICs, vascular interstitial cells.

The mechanism by which lipid oxidation accounts for inflammatory response involves the release of pro-inflammatory mediators produced via either enzymatic (such as prostaglandins and leukotrienes) or non-enzymatic processes (reviewed by [29]). When established, chronic inflammation accounts for VIC activation and differentiation [30,31]. In a healthy aortic valve, tissue resident immune cells consist of macrophages and dendritic cells, while T cells or B cells appear absent. Conversely, calcified aortic valves show a higher presence of both leukocytes and macrophages, suggesting the involvement of both innate and adaptive immunity in aortic valve calcification (Figure 1A; [32]). Most immunity components (macrophages, dendritic cells, mast cells, neutrophils, NK cells, B cells and T cells) seem to play a role especially during preclinical microcalcifications, during which they display pro-angiogenic and pro-osteogenic activity ([33]; for extensive review see Ref. [34]). In particular, histological studies showed that oxidized lipid species activate innate immune response through toll-like receptors (TLRs) and the NF-κB pathway [35]. The expression of TLRs (above all TLR2 and TLR4) on VICs also suggests direct action of ox-lipids in osteogenic differentiation [29]. Moreover, in vitro analysis showed the involvement of NOD-like receptor signaling pathways [36]. Therefore, upon infiltration and stimulation, inflammatory cells (above all macrophages) produce a cocktail of pro-osteogenic cytokines and growth factors (IL-1β, IL-6, IL-8, TNF-α, IGF-1, and TGF-β), as well as proteolytic enzymes (collagenase-1/MMP-1, collagenase-3/MMP-13, gelatinase-A/MMP-2, and gelatinase-B/MMP-9) and cysteine endoproteases (cathepsins K and S), which are responsible for cell transdifferentiation and ECM remodeling, respectively [30]. The highest expression level of M1 markers (such as TNF-α and IL-6) suggests the dominant role of the M1-polarized macrophages in the human calcified aortic valve [37]. When the high level of oxidative stress in such an inflammatory microenvironment exceeds the antioxidant activities, immunity cells could undergo autophagic or apoptotic programming [38]. Immunity cell fragments could also serve as “nucleators” for calcium deposition, further contributing to microcalcification establishment (Figure 1A; [15,30,31]). In a subsequent stage, the progressive evolution towards nodular macrocalcification seems to take place in an anti-inflammatory microenvironment, through pathological ECM remodeling being regulated by VIC-derived myofibroblasts and above all osteoblasts (Figure 1A; [30]).

## 4. Architecture of Extracellular Matrix in CAVD

An abnormal ECM remodeling is a crucial hallmark for CAVD. ECM architecture in healthy aortic valve is assured by cross-talking mechanisms between VICs and VECs. Circulating mesenchymal cells, innate immunity cells and extracellular vesicles release are involved, as well. A healthy aortic valve displays a different ECM composition in the internal layers. The outflow layer of the aortic valve, *fibrosa* displays a high content in type I and III collagen bundles responsible for the mechanical strength. The inflow layer *ventricularis* displays high content in radially oriented elastic fibers, which are responsible for the correct aortic valve closure at the end of diastolic phase of the cardiac cycle, and support mechanical pressure during the alternation of the systolic and diastolic phases. The central layer, *spongiosa*, is rich in proteoglycans (PGs) and glycosaminoglycans (GAGs) which confer the properties of plasticity and resistance to compression to the layer. Moreover, the higher hydrophilicity of the PGs reduces shear between the *fibrosa* and *ventricularis* layers [39].

### 4.1. Dystrophic Calcification and Biomineralization

It is accepted that progressive calcification of ECM observed in CAVD consists of two different mechanisms: dystrophic calcification and biomineralization or ossification [40,41]. According to this view, the first step of aortic valve calcification occurs as osteoblast-independent mechanism, indeed it is also indicated as “diffuse calcification secondary to myofibroblast differentiation of VICs” [15]. Conversely, in a more advanced step, osteoblast activity accounts for calcium crystal organization, as above mentioned. However, the relative contribution of each of these two processes is at present unknown [15].

Microarchitecture alterations in human valves with CAVD have been reported at every phase of CAVD pathogenesis. Regarding dystrophic calcification, it was reported that after endothelium injury, disruption of basement membrane, infiltration of inflammatory cells and early VIC activation, the calcification process started at the leaflet’s hinge, accompanied by abnormal collagen deposition and PG expansion [42]. The resulting valve stiffening takes place concurrently with passive deposition of hydroxyapatite. Microscopic calcified nodules can be observed in proximity of endothelial damage, probably as a result of cell and fiber fragment aggregation in “nodules” (Figure 1A). Inside these nodules, cells undergo apoptosis and ECM fibers are degraded, serving as a substrate for calcium and phosphate deposition [40]. The apoptotic cells to which we refer comprise both myofibroblasts and immunity cells. The mechanism by which myofibroblasts undergo apoptosis has been partially described by [43]. The authors showed that in presence of osteogenic stimuli, VICs cultivated on matrices were not very rigid, defined as “compliant”, survived and differentiated in osteoblast-like cells. Conversely, VICs grown on stiffer matrices differentiated to contractile myofibroblasts and formed calcified aggregates containing apoptotic cells [43]. Moreover, according to [15], myofibroblasts display a greater ability to contract the ECM when cultivated on a harder substrate. These results point to a role for contractile property of myofibroblasts in apoptosis activation. Conversely, the mechanism by which immunity cells undergo apoptosis in the inflammatory aortic valve has been associated to the level of oxidative stress [38].

Afterwards, nodular calcification extends from the middle to the proximal tip regions of the aortic surface of the leaflet [42]. In this phase, the major stiffness of the valve accounts for increased mechanical stress and injury, which results in further calcification and osteoblast differentiation. Then, VIC-derived osteoblasts drive the biomineralization phase, during which calcification mainly occurs through the active secretion of bone matrix. These two processes have also been associated to the two main types of calcification described in clinical studies: “intrinsic” at the hinge of postmortem leaflets with no clinical AS; and “nodular”, primarily found in the middle region of more severely affected postmortem leaflets with clinical AS [42].

### 4.2. The Extracellular Vesicles as Mediators of Aortic Valve Calcification

The molecular mechanisms by which activated VICs, activated VECs and inflammatory cells cooperate to mineralize the ECM are still poor understood. Histological analysis of human calcified aortic valves and in vitro studies suggested that cells secrete extracellular vesicles able to drive mineralization [44,45]. Extracellular vesicles (EVs) display high heterogeneity; however, they can be divided into two main categories: exosomes and microvesicles. The term exosome was initially adopted to refer to membrane vesicles (30–100 nm in diameter) released by reticulocytes during differentiation, as a means of eliminating unneeded compounds from the cell [46,47]. Exosomes rise during maturation of early endosome: internal budding of the endosomal membrane gives rise to intraluminal vesicles and then to multivesicular endosomes, also indicated as late endosome [48]. During this process, many cytoplasmic components can be embedded in endosomes and then directed to either lysosomes or autophasome for degradation or the plasma membrane for secretion [49]. Exosomes are typically 30–150 nm in diameter. Micro-vesicles biogenesis takes place via an initial specific redistribution of plasma membrane protein and lipid components, aimed to regulate membrane rigidity. Then, a direct outward blebbing and pinching of the plasma membrane accounts for the release of micro-vesicles into the extracellular space [50]. The micro-vesicles are between 50 nm and 1000 nm in diameter, but can also be larger in cancer cells.

As pointed out by Mancuso et al., many advances have been achieved in understanding the biological role of extracellular vesicles derived from cardiac resident cells in heart tissue homeostasis and repair [51]. In particular, they underlie the role of exosomes as mediators of the paracrine action of cardiac stem cells, widely recognized for their regenerative and reparative functions. According to most of the literature, exosomes may serve as a tool for disease evaluation and prognosis prediction, and may provide novel clinical biomarkers for new therapeutics.

In the context of CAVD pathogenesis, EVs might mediate the action of immunity cells, circulating mesenchymal cells and resident cells in the exacerbation of the disease (Figure 1A). From a review of the literature we can identify two specific subtypes of EVs which could mediate ECM mineralization: “mineralized spheroid micro particles” and “matrix vesicles”. Mineralized spheroid micro particles (1–3 μm), first described in 2013 have been associated to the microcalcification process occurring during dystrophic calcification [44]. The study of [45] showed that mineralization of VICs cultures is characterized by the presence of “mineralized spheroid micro particles” at the cell surface. Accordingly, similar particles have been observed on the surface of the human calcified aortic valve. Although the mechanisms involved in the secretion of “mineralized spheroid micro particles” have not been described, it was suggested that VIC osteogenic differentiation and apoptosis are associated to their production [49]. This hypothesis further supports the correlation between apoptotic areas and the calcification process [52,53,54].

A different role has been suggested for the release of matrix vesicles (MVs) during CAVD pathogenesis. Initially, MVs were defined as membrane-bound microparticles serving as sites for mineral formation in the cartilage growth plate [55]. Then, the formation and release of MVs in CAVD have been associated with biomineralization [56]. In 2011, Wuthier et al. thoroughly described both the formation and the role of MVs in calcification process, suggesting that “the entities most responsible for the de novo induction of crystalline mineral in most vertebrate hard tissues are MVs” [56]. Regarding the intracellular origin, it was suggested that MVs can derive from lysosomal cytoplasmic vesicles or from peripheral cytoplasmic constrictions and gemmation [57,58]. The latter hypothesis well fits with the identification of MVs at the apical side of microvilli-like membranes in osteoblasts [59]. Despite this evidence, there is no accord on the mechanisms of MV biogenesis. Conversely, it is evident that both the cell source and the specific microenvironment dictate MV biogenesis routes. Although exosomes, microvesicles, and MVs display similar membranous phospholipid bilayer, molecular cargoes appear different [57]. Notably, MVs are enriched in matrix metalloproteinases (MMPs) that probably mediate ECM remodeling (Figure 1B; [60]). It is remarkable the fact that several ectonucleotidases involved in nucleation of calcium and phosphate, such as alkaline phosphatase (ALP), ectonucleotide pyrophosphatase/phosphodiesterase family member 1 (E-NPP 1) and 5′-nucleotidase (5′-NT, also called CD73), are overexpressed in extracellular vesicles [45,61]. According to scientific data, VICs, macrophages, and smooth muscle cells release MVs accounting for biomineralization of the aortic valve [62,63,64,65]. The mineralization process involving MVs has been reviewed by [57]. It was suggested that mineralization observed in CAVD starts in the MVs after they are released from the cell and only after their attachment to the ECM [66]. After MV secretion in the incomplete mineralized osteoid, an influx of Ca^2+^ and PO_4_^3−^ through their membrane transporters and the activation of specific enzymes trigger calcium phosphate nucleation and, consequentially, apatite formation. Crystals of hydroxyapatite progressively increase in number, so the mineralization process continues [67]. On the other hand, different scientific approaches point to an alternative mechanism, suggesting that the mineralization process is initiated in cells prior to the development of mineral foci in MVs or in ECM fibers [68]. It is evident that additional regulating factors, such as the extracellular Ca^2+^ and PO_4_^3−^ content or other inhibitors and promoters of mineralization can modulate MV-mediated calcification [69]. For instance, the presence of calcification inhibitors such as matrix-Gla Protein, fetuin-A and osteoprotegerin in MVs might account for their inactivation during CAVD pathogenesis. Calcified valves have also been known to contain nanoparticles enriched in fetuin-A, albumin or apolipoproteins. Initially indicated as nanobacteria, these nanoparticles have been characterized as particles able to bind ions, nucleic acids, lipids, and carbohydrates, serving as a focus for matrix calcification [7,70].

### 4.3. Production and Distribution of Collagen Fibers and Elastic Fibers in CAVD

Collagen is the most abundant protein in mammals. Many types of cells, such as fibroblasts, osteoblasts, chondroblasts, epithelial cells and so on can produce it. The collagen superfamily comprises 28 members with a common structural feature, i.e., the presence of a triple helix collagens consisting of three polypeptide chains, called α chains, numbered by Roman numerals in vertebrates. The diversity of the collagen family is increased by the existence of several α chains and several molecular isoforms; moreover, hybrid isoforms comprised of a chain belonging to two different collagen types can also be found. Collagen α chains vary in size from 662 up to 3152 amino acids for the human α1 (X) and α3 (VI) chains, respectively [71]. The three α chains can be either identical to form homotrimers (e.g., collagen II) or different to form heterotrimers (e.g., collagen IX). Fibrillar collagens (e.g., I, II, II and V) contain one major triple-helical domain. In contrast, collagens belonging to the fibril-associated collagens with interrupted triple-helices (FACIT), the multiplexins and the membrane collagen subfamilies, contain several triple-helical domains [72]. Some physical and chemical elements account for the stabilization of the triple helix, such as the presence of multiple glycine, proline and hydroxyproline repeats, interchain hydrogen bonds, and lysine and aspartate electrostatic interactions [71]. The collagen matrix of normal aortic valves is mainly formed by type I collagen (70%), with about 25% of type III [73]. Type IV collagen can also be detected. Type I collagen is the predominant component of the *fibrosa* in healthy cardiac valves, while the *spongiosa* and *ventricularis* are mainly composed, respectively, of PGs and a collagen/elastin network (Figure 1A; [74]). CAVD progression starts with valve thickening, evolves with fibrosis and then with calcification, with important implications for valve function. Valve thickening and subsequent fibrosis are caused by an excessive deposition of ECM components including collagen, and result in overgrowth, hardening, and scarring of valvular tissue [75]. One potential mechanism responsible for the excessive collagen production in CAVD involves TGF-β. TGF-β can be released by all cell types and stimulates the formation and deposition of the ECM via canonical Smad signaling in both physiological and pathological conditions. Accordingly, a high level of TGF-β has been shown in fibrotic organs including the stenotic aortic valves [39]. Through the use of Second Harmonic Generation microscopy, by quantifying collagen fiber characteristics, distribution, and crosslinking enzymes, it has been demonstrated that CAVD is associated with specific micro architectural remodeling. In particular, the majority of collagen fiber alterations were found in the *spongiosa*, where the number of collagen fibers increased >2-fold. Fiber width and density also increased accordingly [76]. Conversely, in the *fibrosa*, collagen fibers became significantly shorter, but did not change in terms of number, width, alignment or density. Immunohistochemical staining for lysyl oxidase (LOX), which catalyzes the formation of highly reactive aldehydes from lysyl residues and accounts for collagen cross-linking, showed localized increased expression in the diseased *fibrosa*, suggesting that collagen overexpression might occur. However, the few alterations reported in the collagen network suggest that collagen degradation is overcoming in the *fibrosa* in CAVD, while LOX up-regulation may be responsible for the increased crosslinking of existing fibers. The increased collagen deposition and cross-linking reported in the *spongiosa*, without any change in LOX, suggest a sustained increase in collagen production [76]. In vitro studies showed an inverse relationship between collagen and biomineralization occurring in the advanced CAVD stage [77]. Indeed, removal of collagen in whole organ leaflet cultures was responsible for increased VIC proliferation and differentiation, accordingly to the overexpression of markers associated with myofibroblast and osteoblast activity, such as α-SMA and ALP, respectively. Gene expression analysis also showed the up-regulation of osteocalcin and bone sialoprotein. Finally, these molecular changes result in increased mineralization, as indicated by the positive von Kossa staining and the increased total calcium content [78]. In conclusion, in CAVD, the phase of increased collagen production that leads to valve fibrosis is followed by a phase of increased turnover, which leads to a decrease in the collagen I and III content and ECM remodeling. These phenomena are linked to pathophysiological changes in the structure, function and phenotype of VICs undergoing osteogenic differentiation.

Elastic fibers are physiologically synthesized by fibroblasts, chondroblasts and smooth muscle cells. The production of elastic fibers starts with the synthesis of the precursor proelastin within the cells. Then, the proelastin is cleaved and released in the extracellular space as tropoelastin. In the ECM, elastic fiber organization requires the interaction of tropoelastin with fibrillins 1, fibrillin 2 and fibulin 1. Elastic fibers are produced during both embryonic and postnatal development, while its reduced turnover in adults accounts for the decreased elasticity reported in old age [79]. The lamina *ventricularis* of aortic valves contains a layer of elastic fibers. Moreover, elastic fibers have also been described in the *spongiosa* [80]. Much evidence suggests that elastin can play important roles in the control of calcification [42,81]. For example, in mouse models of Marfan’s syndrome a defect in the fibrillin-1 gene may cause calcification of elastic fibers [82]. In CAVD, progressive increase in elastin fragmentation has been associated with calcification severity [41,83]. Collagen and elastin content in aortic valve commonly decline with age. A similar event has been associated to the up-regulation of both elastin- and collagen-degrading enzymes, e.g., cathepsins and MMP1. The strong up-regulation of both cathepsins (cathepsins S/K/V) and matrix metalloproteinases (MMPs) (MMP-1, MMP-2, MMP-3, MMP-12) reported in CAVD has been indicated as mandatory for valvular remodeling and calcification [84]. As reported above, immunity cells including macrophages strongly contribute to this process. Interestingly, cathepsin inhibitor cystatin C is also elevated in the valve of CAVD patients, while tissue inhibitors of metalloproteinases (TIMPs) appear differentially expressed. In particular, TIMP-1/2 are over expressed whereas TIMP-3/4 are downregulated [84,85]. In conclusion, during the different phases of CAVD progression, alteration of collagen and elastin turnover is shown to be the real trigger of pathological ECM remodeling, regardless of their quantity.

#### 4.3.1. The Role of Hypoxia in Collagen and Elastin Remodeling

What is the triggering factor for MMP and cathepsin up-regulation? Much evidence suggests a role for hypoxia (Figure 1B; [86]). Valve thickening and subsequent fibrosis reported during the early phase of CAVD are accompanied by reduced oxygen availability, contributing to the stabilization of hypoxia inducible factor-1 alpha (HIF-1α) that, in turn, induces a metabolic adaptation through the upregulation of vascular endothelial growth factor (VEGF) and the formation of new vessels. If on one side, the neovascularization restores normoxia, on the other hand it accounts for VIC activation and ECM remodeling, including the formation of calcific nodules. These observations well fit with the high expression of HIF-1α in the calcific nodules of aortic valves, and the consequent activation of different pathways underling ECM remodeling, including NF-κB [87] and MMPs 2 and 9 [86], as well as neutrophil gelatinase-associated lipocalin (NGAL) [57]. NGAL has gained interest for its ability to positively modulate MMP9 activity, accelerating ECM breakdown [88]. In hypoxic aortic valves the activity of the MMP9–NGAL complex is significantly increased. The presence of ectopic elastic fibers in the *fibrosa* suggests altered elastin homeostasis [89]. So, hypoxia play a key role in ECM remodeling by regulating elastin and collagen metabolism.

#### 4.3.2. The Role of the SDF-1/CXCR4 Axis in Collagen and Elastin Remodeling

SDF-1/CXCR4 signaling has been suggested as regulator of angiogenesis in valves (T 1C). SDF-1, also known as CXCL12, is a chemokine with homeostatic functions. It is constitutively expressed in many organs and is associated to tissue damage. Moreover, the CXCL12/CXCR4 pathway has been implicated in tumor metastasis, via the regulation of epithelial–mesenchymal transition (EMT), EndMT and EMT-Like Trans-Differentiation, and has been suggested as useful marker in metastatic risk assessment [90,91,92,93]. In particular, CXCL12 expression in hypoxia exerts direct proangiogenic action, via the recruitment of CXCR4-expressing endothelial progenitor cells [94]. In CAVD, Dorfmüller P et al. showed constant SDF-1 expression in endothelial cells thoughout the valve, while CXCR4 expression was mostly reported in the micro-vessel aggregates next to the nodule [95]. SDF-1/CXCR4 pathway activation in the calcified aortic valve might account for the recruitment of inflammatory elements, via the induction of angiogenesis. This observation fits well with the high IL-6 expression level reported in the calcified valve [95].

### 4.4. Production and Distribution of Glycosaminoglycans and Proteoglycans in CAVD

Despite the well-documented role in tissue structure integrity, GAG and PG involvement in the regulation of cell behavior has been poorly investigated. PG aggregates are a major component of the ECM. Each PG consists of GAGs and proteins. GAGs are linear polymers of disaccharides with sulfate residues. They are composed primarily of glucuronic acid, its epimeriduronic acid, and N-acetylgalactosamine sugar monomers. Such monomers have variable sulfation patterns. Different types of GAGs are attached to a core protein to form a PG. The core protein, in turn, is linked to a hyaluronan molecule via a linking protein. So, the hyaluronan molecule is the axis of a PG aggregate. GAGs control the biological functions of PGs by establishing links with cell surface components, growth factors and other ECM constituents [79]. To date, more than 40 genes encoding different PGs have been identified, which have been organized in four major classes on the basis of cellular and subcellular localization [96]. Among them, only few PGs have been investigated for a role in CAVD pathogenesis and progression [97,98,99,100,101,102].

In general, during CAVD progression PGs and GAGs act by retaining water, TGFβ, lipids, which attract inflammatory cells and cause calcification [103]. Some evidence has showed that PGs versican, decorin, and biglycan, as well as the GAG hyaluronic acid (HA) are abundant within the tissue immediately surrounding the calcified nodules, but absent from the nodules. Versican and HA are also abundant in the vicinity of the larger nodules, but less so in the smaller “prenodules” [104].

In CAVD, both distribution and homeostasis of some hyalectans, namely aggrecan and versican appeared altered. The increased expression of both MMPs and “A disintegrin-like and metalloprotease domain with thrombospondin type 1 motifs” (ADAMTS) proteases in aortic valve accounts for the cleavage of aggregan in peptides able to stimulate TLRs, as recently reported in the arthritic joint [105]. Interestingly, while the expression of specific MMPs increases in CAVD, ADAMTS seem to be down-regulated [106]. It was reported that ADAMTS5 down-regulation, above all at the aortic side of the valve, accounts for VIC activation and myofibroblast or osteoblast transdifferentiation [106]. Indeed, ADAMTS5 down-regulation determines aggrecan accumulation which, in turn, might alter VICs’ phenotype as previously reported for vascular smooth muscle cells [98].

Recent studies showed that some components of the largest class of proteoglycans, the so-called small leucine-rich proteoglycans (SLRPs), play a key role in CAVD pathogenesis. In healthy aortic valve, SLRPs are abundant and mostly located in the inner part of the valve [107]. Histological analysis showed that the fibrotic and calcified aortic valve display increased levels of SLRPs, especially biglycan, via a mechanism that involves the upregulation of the TGFβ-pathway [101]. Biglycan overexpression, in turn, activates VICs by binding to TLR2, and induces the release of phospholipid transfer protein (PLTP), one major protein involved in modifications of lipoproteins [108].

Conversely, in vitro studies showed that osteogenic differentiation and mineralization of VICs are characterized by a reduced expression of Asporin, also known as periodontal ligament-associated protein1 (PLAP1), suggesting an inhibitory role for this PG in the aortic valve [99]. In this case, mineralization associated to Asporin downregulation seems to involve the activation of Wnt/*β*-catenin signaling. Another in vitro study showed that, beside Wnt/*β*-catenin signaling, Asporin also inhibits osteogenic differentiation via direct binding to BMP and consequent inhibition of BMP-Smad1/5/8 signaling in periodontal cells [109]. Interestingly, the small leucine-rich PGs decorin, biglycan, and lumican are evenly distributed in normal human aortic valves, yet frequently co-localized with collagen and play a role in collagen fibrillogenesis. So, a role in fibrosis reported in CAVD progression is also conceivable for such molecules [104]. Accordingly, in [102] the authors showed that the amount of lumican was decreased in the thickened and calcified areas of AS aortic valves, suggesting its involvement in both the impairment of the collagen network and inflammatory events.

An altered expression of proteoglycan 4, also known as lubricin, has been recently associated to fibrosis and mineralization occurring in the aortic valve [102]. Although the proteoglycan CD44 in the context of CAVD pathogenesis has been less investigated in the past, in the last few years numerous studies have pointed to a key role in mineralization. CD44 is able to interact with many ECM components including collagen, fibronectin, laminin, serglycin, MMPs and chondroitin sulfate [110]. Moreover, CD44 mediates osteopontin-mediated calcium deposition in human VICs culture, resembling the calcification process in the early stage of CAVD [111]. In normal tissues, CD44 represents the major HA receptor, so it mediates most of the HA function in the valve. Accordingly, targeting of CD44 results in the disruption of HA metabolism, wound healing, and keratinocyte proliferation [112]. HA comprises ~35% of the total GAGs in the aortic valves and its content has been shown to increase with age, evidence that fits well with increased prevalence of CAVD in elderly patients [107]. Regarding the molecular mechanisms of HA in CAVD, it was suggested that higher expression of HA might account for the activation of BMP-mediated osteogenic differentiation. This effect seems to be achieved via a direct binding of HA to the “protein product of TNF-α stimulated gene-6” (TSG-6), recognized as an inhibitor of BMP2-mediated osteoblast differentiation. Indeed, TSG-6 plays a role as potent HA cross-linking agent [113]. In this scenario, when high expression of HA is established, a major fraction of free BMP2 can be available for the triggering of the calcified nodule development. Obviously, in valve regions where hyaluronan synthase (Hyal-1) expression is strong, the HA is more likely degraded and unable to bind to TSG-6 [114]. The role of HA in calcification has also been shown in vitro. In particular, rat VICs grown on HA polyacrylamide gels showed a significant increase in the size of the nodules as compared with VICs grown on collagen polyacrylamide gels, suggesting that HA has a direct effect on mineralization. Moreover, siRNA knockdown of CD44 reduced mineralization on HA polyacrylamide gels [115].

### 4.5. Production and Distribution of Tenascin-C and Periostin in CAVD

The so-called matricellular proteins were previously identified as transient, rather than constitutive components of the ECM [116]. They are incorporated into the ECM of remodeling tissues in cancer growth, wound healing, and in response to injury and stress, especially in fibrotic ECM, and during development. Matricellular proteins can bind growth factors such as VEGF, basic fibroblast growth factor, and (latent) TGF-β and can modulate the actions of these factors [117]. These proteins are also capable of mutual interactions and bonds. Among them, Tenascin-C (TNC) and periostin (PO) are coordinately induced by mechanical stress and have been recognized as important mediators of persistent tissue fibrosis in CAVD.

TNC is a hexameric ECM protein with several molecular forms which are produced by alternative splicing and protein modifications. In the embryo, TNC is found around motile cells in morphogenesis, and in connective tissues, such as bone, cartilage and in the central nervous system. In the adult human organism, TNC expression is found only in a few connective tissues that bear tensile strength, underneath some epithelia and in stem cell niches—such as the crypts of the intestine, bulge of hair follicles and bone marrow [118,119,120]. TNC interacts with other ECM components: fibronectin, collagen, PO and fibrillin-2 as well as PGs of the lectican family and perlecan.

In normal aortic valves, TNC expression is associated with the basement membrane beneath the endothelial cells, whereas stenotic valves show immunoreactivity in the deeper layers of the valves, especially in calcific nodules. The main sources of TNC production in diseased valves are stromal myofibroblasts [121]. Moreover, TNC can bind to several integrins, including α_2_β_1_, and α_v_β_3_ that have been demonstrated to be involved in neovascularization [122]. Although healthy human valves are avascular, the calcified leaflet showed vascularization foci, whose entity was proportional to the disease stage. So, TNC has been indicated as mediator of neovascularization, during which the modulation of inflammatory infiltrate involves the direct interaction of TNC with macrophages and lymphocytes [121,123]. Another mechanism involves the production of MMP-2. Indeed, TNC accounts for the upregulation of both MMP-2 expression and its collagenolytic activity, the degradation of the subendothelial basement membrane and then the formation of a new channel available for angiogenesis [124]. Some studies revealed a decreased expression of tenascin-X in calcified aortic tissue, suggesting its involvement in ECM remodeling [125,126].

PO was identified in 1993 as a putative cell adhesion protein [127]. It is related to the fasciclin-1 family and is strongly expressed in collagen-rich fibrous connective tissues subjected to mechanical stress, such as periodontal ligament, endocardial cushions, mature cardiac valves and cardiac fibrous skeletal tissue [128]. Alternative splicing of the C-terminal sequence gives rise to four PO isoforms which display a tissue-dependent expression pattern [128]. PO is an inducer of fibrillogenesis and directly or indirectly interacts with type I collagen, fibronectin, tenascin C, and BMP1 [129]. The molecular mechanism of PO action in collagen cross-linking includes an enhancement of proteolytic activation of LOX [130].

The constitutive expression of PO in the heart valves, from embryo to adult, indicates its key role not only during development but also in tissue repair. According to the “periostin hypothesis”, during development PO promotes differentiation of prevalvular mesenchyme into collagen-producing fibroblastic cells termed VICs; however, we can also assert that PO inhibits differentiation of cushion cells into non-fibroblastic lineages (e.g., osteoblastic or chondrogenic cells) [131]. Regarding the role of PO in heart disease, its involvement in coronary artery disease, hypertension, cardiac valves diseases and cardiac fibrosis from various causes has been suggested. In particular, during organ damage and regeneration, overproduction of PO is associated to pathological fibrosis and impairment of organ function. Thus, PO has become a main target in understanding the mechanism of fibrosis [132]. The role of PO in calcification is still not clear, but most of the evidence suggests that a lack or decreased expression of PO is procalcific, accounting for derepression of the osteogenic potential of mesenchymal cells and calcium deposition [133]. Regarding the molecular mechanisms regulated by PO in CAVD, some in vivo studies showed the involvement of Notch signaling [133]. Accordingly, knockout of PO in mice results in the suppression of Notch1 signaling and the induction of several osteogenic factors like RUNX2, which lead to aortic valve calcification [133]. As a matter of fact, the expression of PO is higher on the ventricular side of the valve than on the aortic side, where calcification occurs [134]. In humans, expression of PO is lower in VICs from calcified than from healthy valves and, after stimulation with lipopolysaccharides (LPS), the expression of PO is decreased in cells cultured on collagen [134].

In summary, PO and TNC play an important role in the regulation of the ECM in AS. Their mechanism of action seems to also involve reciprocal interactions. Furthermore, they certainly influence phenomena related to valvular disease such as neoangiogenesis and fibrosis, as previously reported in other pathological conditions [123].

## 5. Discussion and Future Perspectives

CAVD is not a passive process of aging and calcific degeneration, but it is now widely recognized as an actively and finely regulated process in which valve calcification results from endothelial damage, lipid accumulation and inflammation, similarly to early lesions in atherosclerotic heart disease. The calcific process that characterizes the progression from aortic sclerosis to histopathological and clinical AS is still poor understood, and there is no therapy preventing the disease progression. However, it is clear that ECM components play a pivotal role [135,136]. Valvular fibrosis and the consequent calcification are not only due to an excessive release of matrix components, but to a fine ECM remodeling, which evolves from a passive event to an active one, primarily guided by VICs, VECs, circulating mesenchymal cells, MVs and innate immunity cells. In turn, altered components of the matrix can trigger changes in this plethora of different cell types [137]. In this context, we can hypothesize a role for macromolecular crowding, a biophysical phenomenon based on the principles of the excluded-volume effect [138]. In particular, biological systems are dominated in vivo by macromolecular crowding, for which the intracellular environment is characterized by a dense and highly diverse pool of macromolecules at different concentrations and no macromolecule at high concentration. These macromolecules, by occupying a substantial part of the available volume, reduce the accessible volume favoring macromolecule assembly, protein folding and enzymatic activity [139]. The ECM remodeling observed in CAVD, characterized by dysregulated and ectopic expression of most of its components, could alter macromolecular crowding driving cell trans-differentiation. Although the influence of macromolecular crowding on both collagen maturation and chondrogenesis and osteogenesis differentiation of human bone marrow stem cells has been showed in vitro [140], no data have been reported in cell activation observed in CAVD.

In CAVD patients, at present surgical valve replacement represents the standard treatment option. Valve replacement consists of the application of either mechanical valves or bioprosthesis. Mechanical valves are prepared by using synthetic materials, such as titanium. They are free from calcification, have a duration of about twenty years but require anticoagulant therapy due to the risk of thrombus and clots forming on their surface. On the other hand, bioprosthetic valves (including xenografts and allografts) display no thrombogenic activity and a variable propensity to mineralization, with a shorter duration. In the few last years, a different approach has been developed, in order to reproduce a nearly normal valvular structure via the application of bioengineered valves. The most important advantages of bioengineered valves consist of low propensity to mineralization and high hemodynamic performances. Bioengineered valves can be obtained from acellular non-valvular scaffolds or decellularized native valves, which can be repopulated with host cells [99,141]. In addition, scaffold matrices may also consist of artificial matrices synthesized from synthetic or natural polymers. The latter have been further classified as porous, fibrous, and hydrogel scaffolds [142]. Among them, matrices synthesized from the ECM constituents of native valve (collagen, elastin and GAGs) such as hydrogel have been analyzed in vitro with good results [143,144]. Hydrogels derive from natural components, such as fibrin, collagen, GAGs and gelatin, host cell-binding antigens useful for a suitable cell repopulation, and display signaling functions, because of their capability to hold back various active molecules. Moreover, synthetic hydrogels can be modified by the addition of cell-binding motifs, such as Arg-Gly-Asp (RGD) for integrin binding. Mediators promoting cell differentiation can be also inserted within the hydrogel at specific concentrations. Moreover, in order to confer the material strength indispensable for heart valve functioning, various approaches have been used, such as the adding of synthetic methacrylate groups modulating the stiffness of the material [142]. It is important to underline that all these modifications are able to condition the phenotype of the cells populating the scaffold. After cell-seeding, bioengineered valves require further mechanical conditioning, aimed to activate cell proliferation and differentiation, allow ECM remodeling, and confer adequate mechanical properties [142]. Moreover, in this context, the investigation of the ECM alterations in CAVD could provide us with important insights for the development of reliable devices including reconstituted matrices.

The complexity and slow development of CAVD over time can be considered an opportunity regarding the possibility to plan therapeutic interventions before surgery becomes inevitable. In fact, they provide time and multiple targets to exploit, but it is clear that any attempt at drug therapy must be directed to the prevention of ECM alterations. Today, medical management of patients with CAVD comprises different class of drugs such as lipid-lowering drugs, including statins and proprotein convertase subtilisin/kexin type 9 (PCSK9) inhibitors. In particular, the different clinical trials conducted in order to verify the efficacy of statins in halting CAVD progression or preventing the onset, have suggested that the efficacy of the treatment is dependent on the stage of CAVD [145]. Inhibitors of the renin–angiotensin–angiotensinogen system, mineralocorticoid receptor antagonists, modulators of nitric oxide pathway and anti-calcific agents (vitamin K, bisphosphonates and denosumab) have been also suggested for CAVD therapy, however their efficacy requires more investigation [146]. Recently, Donato M et al. in their interesting work summarize the new emerging targets for CAVD therapy [146]. In addition, recent evidence has suggested that epigenetic markers such as miRNAs or lncRNA [11] can be implicated in the landscape of phenotypical changes occurring in CAVD. Furthermore, the microbiome, an essential player in several diseases, including the cardiovascular ones, has recently been linked to the inflammation process occurring in CAVD [147]. However, their potential role as therapeutical targets needs further investigation. Finally, although the contribution of ECM components to CAVD pathogenesis has been partially described, no specific therapeutic approaches have been defined yet.

We can assume that given the difficulties in blocking or reversing the disease at an early stage, the most important contribution that can derive from the knowledge of the structure and dynamics of the ECM in CAVD, is a reconstructive approach alongside surgery. Indeed, the intracellular pathways that lead to the synthesis of the ECM components or enzymatic activities responsible for its remodeling could be modulated via pharmacological approach. In many other pathologies, this approach is already possible; therefore, the study of the architecture and dynamics of the ECM in CAVD can be a field of research leading to new therapeutic possibilities. Among the native ECM molecules or ECM-like materials available for clinical purposes, HA has been used for a long time. HA has been recognized as a key element for healthy valve function. HA depletion, in both two-dimensional (2D) and 3D VICs culture, is sufficient to induce pathological outcome regulating the phenotype and calcification of VICs [148]. In a recent model, HA enrichment induces VIC secretion of VEGF while chondroitin sulfate enrichment leads to increased deposition of oxidized lipoproteins, which, in turn, induce myofibroblastic activation and the production of inflammatory cytokines [149]. In tissue engineering and regenerative medicine, HA has been used in combination with growth factors, cells, nanotechnology, and advanced scaffolds [150]. Moreover, HA represent a natural macromolecular crowder able to increase cell-mediated ECM deposition in vitro [151].

In conclusion, both the ECM components and the different cell types appear attractive therapeutic targets for CAVD, however specific targeting could account for considerable side effects. So, it is important to define the optimal therapeutic approach in order to restore both the histological structure and the normal function of the valve, reducing the number of patients requiring aortic valve replacement.

## Data Availability

Not applicable.

## Abbreviations

ADAMTS	A disintegrin-like and metalloprotease domain with thrombospondin type 1 motifs ALPAlkaline Phosphatase
AS	aortic stenosis
BMP	bone morphogenetic protein
CAVD	calcific aortic valve disease
ECM	extracellular matrix
EMT	epithelial-mesenchymal transition
EndMT	endothelial-mesenchymal transition
EVs	extracellular vesicles
GAGs	glycosaminoglycans
HA	hyaluronic acid
HIF-1α	hypoxia inducible factor-1 α
ICAM-1	intercellular adhesion molecule 1
LOX	lysyl oxidase
LPS	lipopolysaccharides
MMPs	matrix metalloproteinases
Msx2	MshHomeobox 2
MVs	matrix vesicles
NGAL	neutrophil gelatinase-associated lipocalin
PCSK9	proprotein convertase subtilisin/kexin type 9
PGC-1α	peroxisome proliferator-activated receptor-γ coactivator 1-α
PGs	proteoglycans
PO	periostin
RHAMM	receptor for hyaluronate-mediated motility
RUNX2	runt-related transcription factor 2
SMCs	smooth muscle cells
TAVI	trans-catheter aortic valve implantation
TAVR	trans-catheter aortic valve replacement
TGF-β	transforming growth factor-β
TIMPs	tissue inhibitors of metalloproteinases
TNC	tenascin-C
TSG-6	TNF-α stimulated gene-6
VECs	vascular endothelial cells
VEGF	vascular endothelial growth factor
VICs	vascular interstitial cells
α-SMA	α-smooth muscle actin
